# A Cross-Sectional Study of Barriers to Personal Health Record Use among Patients Attending a Safety-Net Clinic

**DOI:** 10.1371/journal.pone.0031888

**Published:** 2012-02-20

**Authors:** Joan F. Hilton, Lynsey Barkoff, Olivia Chang, Lindsay Halperin, Neda Ratanawongsa, Urmimala Sarkar, Yan Leykin, Ricardo F. Muñoz, David H. Thom, James S. Kahn

**Affiliations:** 1 Department of Epidemiology and Biostatistics, University of California San Francisco, San Francisco, California, United States of America; 2 Department of Medicine, University of California San Francisco and HIV/AIDS Division, San Francisco General Hospital, San Francisco, California, United States of America; 3 Department of Psychiatry, University of California San Francisco, San Francisco, California, United States of America; 4 Department of Family and Community Medicine, University of California San Francisco at San Francisco General Hospital, San Francisco, California, United States of America; Texas Tech University Health Sciences Center, United States of America

## Abstract

**Background:**

Personal health records (PHR) may improve patients' health by providing access to and context for health information. Among patients receiving care at a safety-net HIV/AIDS clinic, we examined the hypothesis that a mental health (MH) or substance use (SU) condition represents a barrier to engagement with web-based health information, as measured by consent to participate in a trial that provided access to personal (PHR) or general (non-PHR) health information portals and by completion of baseline study surveys posted there.

**Methods:**

Participants were individually trained to access and navigate individualized online accounts and to complete study surveys. In response to need, during accrual months 4 to 12 we enhanced participant training to encourage survey completion with the help of staff. Using logistic regression models, we estimated odds ratios for study participation and for survey completion by combined MH/SU status, adjusted for levels of computer competency, on-study training, and demographics.

**Results:**

Among 2,871 clinic patients, 70% had MH/SU conditions, with depression (38%) and methamphetamine use (17%) most commonly documented. Middle-aged patients and those with a MH/SU condition were over-represented among study participants (N = 338). Survey completion was statistically independent of MH/SU status (OR, 1.85 [95% CI, 0.93–3.66]) but tended to be higher among those with MH/SU conditions. Completion rates were low among beginner computer users, regardless of training level (<50%), but adequate among advanced users (>70%).

**Conclusions:**

Among patients attending a safety-net clinic, MH/SU conditions were not barriers to engagement with web-based health information. Instead, level of computer competency was useful for identifying individuals requiring substantial computer training in order to fully participate in the study. Intensive on-study training was insufficient to enable beginner computer users to complete study surveys.

## Introduction

Electronic medical records provide clinicians with access to health information at the “point of care” [1], [2], [3]. Analogously, Internet-based personal health records (PHRs) provide patients with the ability to access their own health information at their “point of need” [3]–[8]. It is hoped that improved access to health information offered by the PHRs will contribute to enhanced health knowledge, engagement within the health system, personal empowerment and eventually to improved health outcomes. Of concern is whether these advantages are equally available to all patients who may potentially benefit from this technology [9]. Specifically, can those without consistent Internet access, with limited computer proficiency, with mental health challenges or addictions to substances make use of an Internet-based personal health record system? As PHR use expands, the “digital divide” between computer/Internet users and non-users may exacerbate health disparities [10]–[13]. Prior studies have demonstrated racial/ethnic and literacy-related disparities in choice of media for health information among individuals with chronic diseases [9], [14], [15]. Those with mental health diagnoses and substance use conditions (“MH/SU conditions”) experience especially poor health, coupled with diminished capacity to respond to their own health needs.

Mental health disorders are commonly encountered in the primary care setting and are often underdiagnosed [16]–[20]. Mental health diagnoses add complexity to health care, especially among the poor or disenfranchised who typically receive primary care services in the safety net setting [21], [22], [23]. Substance use is also underreported and frequently not identified, leading to its undertreatment [11], [24]–[28]. Online health tools provide an innovative approach to engaging persons with these often-stigmatized conditions. An online PHR facilitates enhanced engagement by providing a means of viewing one's secure personal health information (including diagnostic history, medications, and laboratory findings) and accessing related explanations at the convenience of the patient. Additionally, it can provide appointment reminders and other services that may improve communication among clinicians, patients and their community [29], [30], [31].

To determine if a mental health or substance use (MH/SU) condition is a barrier to engagement with web-based health information we examine baseline data collected via a randomized controlled trial (RCT). We evaluate whether patients with MH/SU conditions, given access to individualized accounts, are less willing or less able to engage than patients without these conditions. Our results may guide efforts to reach out to patients with these vulnerabilities in order to help them make use of health information technologies.

## Methods

Individualized PHRs were developed for patients receiving care at the adult HIV/AIDS Clinic at SFGH [32]. The PHR populates automatically in real time with selected data from the clinician's entries in the patient's Electronic Medical Record (EMR). Any HIV/AIDS-infected patient who received primary care at this Clinic between July 2009 and June 2010 and provided written informed consent was eligible for the parent RCT. The goal of this trial was to assess the effect on HIV-related health outcomes of web-based access to personal (PHR) or general (non-PHR) health information via individualized web-based accounts.

### Recruitment, Consent and Enrollment

During clinic sessions, Research Assistants approached patients individually in the clinic's waiting room, provided them with a general overview of the study's goals and requirements, and invited them to participate. Patients were advised that they would be asked to complete web-based study surveys at 0, 6, and 12 months' follow-up and would be compensated five dollars in gift certificates to a major grocery chain. A Research Assistant met individually with each participant in a private office to complete the enrollment process and to obtain consent.

### Minimal Instruction in Computer Use and Internet Access

Given the safety-net setting of the RCT, Research Assistants helped participants obtain a health information account, explained general and study-specific aspects of a web-based health information account, and offered rudimentary training in computer use – such as how to use a mouse, how to “click and drag,” how to open an Internet browser, and how to complete the surveys embedded within the account. For subsequent access, participants were referred to public libraries and other locations that offer free Internet and computer use where they could complete their study-related surveys.

### Enhanced Instruction in Computer Use and Internet Access

A need to measure computer skills was not anticipated prior to the RCT. However, during accrual months 1–3, Research Assistants noted wide variation in participants' computer abilities and began to record each participant's level of computer competency (beginner, intermediate, or experienced). Study procedures were modified during accrual months 4–12 to have Research Assistants coach participants to complete baseline surveys at the clinic during their initial web-portal access, without guiding responses or breaching confidentiality, and to record the time they spent helping the participant.

### Data Collection and Analysis

Our two dichotomous outcomes, RCT participation by clinic patients and survey completion by RCT participants, were extracted from clinic records and study participation computer logs. They corresponded with distinct research questions and were analyzed separately. In both cases, the primary variable of interest was presence/absence of a MH and/or SU condition. MH and SU conditions were diagnosed and recorded by primary care providers in the EMR; nicotine use was not studied. When these conditions were not recorded, we assumed they were absent.

Among clinic patients, we addressed “willingness to engage” via consent (yes/no) to participate in a randomized trial that provided access to health information via individualized web-based accounts. We used a multivariable logistic regression model to estimate the association between participation in the RCT and MH/SU status, adjusted for demographic characteristics. We analyzed age in decades, combining individuals 60 and older.

Among randomized participants, we addressed “ability to engage” via completion (yes/no) of all nine baseline surveys embedded in the web-based accounts. We used a multivariable logistic regression model to estimate variation in survey completion by MH/SU status, on-study training in computer use (two levels), computer competency (four levels), and demographic characteristics. To identify differential training benefits, we included in the model interactions of training strategy with MH/SU status and with level of computer competency. We summarize these effects via odds ratios (OR), 95% confidence intervals (CI), and likelihood ratio (LR) p-values.

This study was approved by the University of California San Francisco Committee for Human Research (#0833272) and the Privacy Board of the San Francisco Department of Public Health. The randomized controlled trial was registered at clinicaltrials.gov, Registry No. NCT00972348.

## Results

### “Willing to engage” with web-based health information

**Figure 1:**
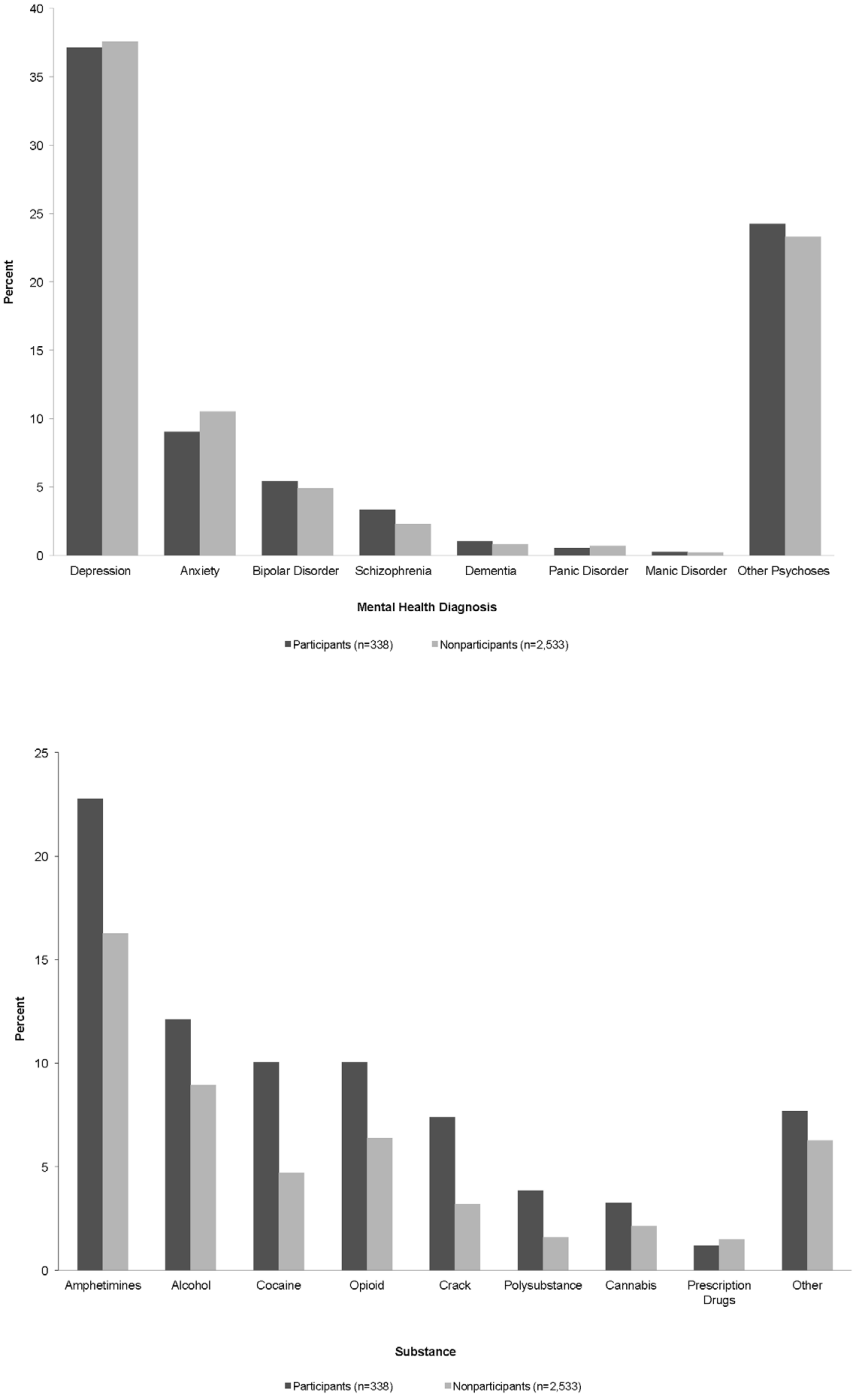
Prevalences of (a) mental health and (b) substance use conditions among study participants and nonparticipating HIV/AIDS clinic patients.

Eligible clinic patients (N = 2,871) predominantly were Caucasian (49%) or African-American (25%) males (84%), with a median (IQR) age of 46 (39–53) years (Table 1). A MH diagnosis was recorded for 58% of patients, with depression being the most common diagnosis; substance use was recorded for 38%, with methamphetamine being the most common substance (Figure 1). Both conditions were recorded in 10% of patients.

**Table 1:** Distributions of mental health/substance use and demographic characteristics of study participants and nonparticipants.

	Participants	Nonparticipants	OR (95% CI)	LR
	N = 338	N = 2,533		p-value
**MH condition**	231 (68%)	1,448 (57%)	1.30 (1.01–1.68)	.043
**SU condition**	179 (53%)	922 (36%)	1.62 (1.28–2.06)	<.0001
**Age (years)**				.0076
20–29	12 (4%)	190 (8%)	0.96 (0.42–2.17)	
30–39	57 (17%)	487 (19%)	1.69 (0.90–3.20)	
40–49	153 (45%)	967 (38%)	2.14 (1.18–3.89)	
50–59	103 (30%)	679 (27%)	1.93 (1.05–3.56)	
≥60	13 (4%)	210 (8%)	1.00	
**Gender**				.22
Men	265 (78%)	2,145 (85%)	1.0	
Women	59 (17%)	303 (12%)	1.30 (0.94–1.80)	
Transgender	14 (4%)	71 (3%)	1.34 (0.73–2.44)	
**Race**				.093
Caucasian	161 (48%)	1245 (49%)	1.0	
African-American	109 (32%)	615 (24%)	1.25 (0.94–1.65)	
Asian	10 (3%)	139 (5%)	0.64 (0.33–1.26)	
Other	58 (17%)	534 (21%)	1.41 (0.81–2.46)	
**Hispanic**				.067
No	275 (83%)	1674 (75%)	1.0	
Yes	58 (17%)	561 (25%)	0.60 (0.34–1.06)	
Unknown	(N = 5)	(N = 298)	–	

To achieve the goals of the RCT we recruited 338 participants. Patients who were not approached or declined were classified as nonparticipants. More participants were aged 40–59, while more nonparticipants were younger or older (p = 0.0063). The gender, race and ethnicity distributions were similar. Adjusted for demographic differences, both MH and SU conditions were more prevalent among participants (OR, 1.30 and 1.61, respectively). Thus we subsequently analyzed combined MH/SU status (adjusted OR, 1.68 (95% CI, 1.24–2.26); p = 0.0004).

### “Able to engage” with web-based health information

Of 338 participants, 70% completed all nine surveys and 17% completed none. The majority of participants were recruited during the enhanced training period (62%). Of 262 participants whose computer competency was assessed, most were categorized as experienced (51%), 29% as intermediate users, and 21% as beginners.

Based on a multivariable model, the survey completion rate was statistically significantly higher among those receiving enhanced training (OR, 2.68 (95% CI, 1.06–6.73); p = 0.032). It also varied by level of computer competency (p = 0.0091) and was elevated among those with intermediate computer competency, relative to beginners (3.99 (1.61–9.93); Table 2). However, in contrast to our expectation that MH/SU conditions are barriers to engagement with web-based health information, we observed a nonsignificant trend toward greater survey completion by those with MH/SU conditions (OR, 1.85 (0.93–3.66); p = 0.076). After adjusting for the effects of MH/SU condition, computer competency, and enhanced training, survey completion was not associated with any demographic characteristic.

**Table 2:** Adjusted associations of survey completion with MH/SU status, training strategy, and level of computer competency.

	Completion rate	Adjusted	LR
		OR (95% CI)	p-value
**Overall**	70% (235/338)	–	
**MH/SU condition**			
Absent	64% (40/62)	1.0	0.076
Present	71% (195/276)	1.85 (0.93–3.66)	
**Accrual period/Training strategy**			0.032
Earlier/Minimal	60% (78/129)	1.0	
Later/Enhanced	75% (157/209)	2.68 (1.06–6.73)	
**Computer competency**			0.0091*
Beginner	48% (26/54)	1.0	
Intermediate	83% (62/75)	3.99 (1.61–9.93)	
Experienced	78% (104/133)	3.44 (1.51–7.81)	
Not assessed	57% (43/76)	1.67 (0.42–6.68)	

*Includes participants not assessed (3 df).

We found that those without MH/SU conditions benefitted from the enhanced training (5.80 (1.44–23.3)) but those with MH/SU conditions did not (1.23 (0.53–2.85)). This contrast (p = 0.022) appeared to be due primarily to low and high completion rates, respectively, in those recruited during the earlier period (Table 3). The benefit of enhanced training did not vary significantly by level of computer competency (p = 0.79); in particular, beginners experienced no benefit (1.77 (0.45–6.98)).

**Table 3:** Adjusted effects of enhanced training, by MH/SU status and levels of computer competency.

Accrual period/Training strategy				
	Earlier/	Later/	Adjusted	LR
	Minimal	Enhanced	OR (95% CI)	p-value
**Overall**	60% (78/129)	75% (157/209)	2.68 (1.06–6.73)	0.032
**MH/SU condition**				0.022
Absent	32% (6/19)	79% (34/43)	5.80 (1.44–23.3)	
Present	65% (72/110)	74% (123/166)	1.23 (0.53–2.85)	
**Computer competency**				0.79*
Beginner	46% (6/13)	49% (20/41)	1.77 (0.45–6.98)	
Intermediate	72% (13/18)	86% (49/57)	4.55 (1.12–18.4)	
Experienced	72% (18/25)	80% (86/108)	2.42 (0.82–7.19)	
Not assessed	56% (41/73)	67% (2/3)	2.62 (0.22–31.4)	

*Includes participants not assessed (3 df).

During the enhanced training period, the median (IQR) minutes of PHR training required by beginner, intermediate and experienced users were 42 (30–49), 29 (20–35) and 15 (13–20), respectively, with no variation by MH/SU status. Participants with advanced computer competencies tended to complete all surveys if they completed any (at least one: 93%; all: 80%), whereas many beginner users did not (at least one: 68%; all: 48%).

## Discussion

We studied initiation of use of web-portals providing health information by patients attending a safety-net HIV/AIDS clinic. We found that having an MH/SU condition was not a barrier to willingness or ability to engage in Internet-based delivery and collection of health information. MH/SU conditions had no effect on the mean duration of on-study computer training or the rate of survey completion. Thus these conditions were not debilitating, with respect to study outcomes, relative to the milieu of challenges encountered by patients who attend safety-net clinics. Patients with MH/SU conditions were over-represented among our participants, relative to the clinic population. Such patients may have been present at the clinic more often or may have been more interested in the study – perhaps compelled by the small monetary inducement to participate.

On average, we were able to significantly increase survey completion rates by offering one-on-one coaching. However, patients with only rudimentary computer competencies at enrollment −21% of our sample – showed little improvement and we conclude that the survey burden – its quantity and/or its complexity – was too high for beginner computer users. We plan to tailor future technology interfaces more closely to strata within the target population. More generally, however, greater investment in training may ultimately be as critical an element for engagement in technology dissemination as access to the technology.

We believe that when patients access health information on the Internet, they experience increased empowerment to play a more active role in their own care, and that this level of engagement is a critical element for technology dissemination. In particular, participants with intermediate computer competency at enrollment showed the greatest benefit from on-study computer training. Acquiring Internet navigation skills could also impact patients in ways that are not anticipated. For instance, newly-acquired computer skills may present opportunities for these patients to access online health management modules, or to access information regarding housing, food security employment and other critical health care issues that could indirectly improve aspects of their health [21], [22], [32], [33].

There are several potential limitations to the generalizability of our findings. First, restriction of the outcomes to study participation and to completion of baseline surveys does not address sustained interest in accessing web-based health information or longer-term effects of the on-study computer training we provided. Analyses of PHR logs corresponding with follow-up time-points would provide such information. Second, although training of participants by Research Assistants significantly boosted the rate of survey completion, many sites may not be able to afford PHR training by highly qualified staff assigned to enable PHR use. Further, the prevalence of intermediate-plus computer competency in our participants (79%) may be higher than in nonparticipants, and higher than in other areas of the United States. The need for quality training, being an essential aspect of technology acceptance and project dissemination, would likely represent a significant financial concern and a potential economic burden. It is possible that using peer trainers or volunteers with some computer and Internet knowledge to engage and train inexperienced users could reduce the costs of training. Finally, the desire and ability to engage in access of web-based health information may differ in populations bearing co-morbidities other than HIV/AIDS and MH/SU.

In this study we demonstrated that patients with HIV/AIDS receiving care in a safety-net setting are willing and able to interact with a web-based health information portal, given the opportunity. An unexpected finding was that patients with MH/SU conditions participated in the study at a higher rate than their counterparts and did not require more time to register for a PHR account or to complete study surveys than individuals without these conditions. Higher levels of both pre-study computer competency and on-study training significantly increased the rate of completion of study surveys. These factors can guide both eligibility criteria and intervention strategies of trials collecting patient-level data via online tools. The lessons learned in this study could help improve PHR acceptance and accelerate the implementation of this technology among individuals often excluded from health technology dissemination.
